# Single dose of a replication-defective vaccinia virus expressing Zika virus-like particles is protective in mice

**DOI:** 10.1038/s41598-021-85951-7

**Published:** 2021-03-22

**Authors:** Brittany Jasperse, Caitlin M. O’Connell, Yuxiang Wang, Paulo H. Verardi

**Affiliations:** 1 Department of Pathobiology and Veterinary Science and Center of Excellence for Vaccine Research, College of Agriculture, Health and Natural Resources, University of Connecticut, Storrs, CT 06269 USA; 2 Present Address: Department of Microbiology and Immunology, School of Medicine, University of North Carolina at Chapel Hill, Chapel Hill, NC USA

**Keywords:** Vaccines, Pox virus, Viral infection

## Abstract

Zika virus (ZIKV), a flavivirus transmitted primarily by infected mosquitos, can cause neurological symptoms such as Guillian–Barré syndrome and microcephaly. We developed several vaccinia virus (VACV) vaccine candidates for ZIKV based on replication-inducible VACVs (vINDs) expressing ZIKV pre-membrane (prM) and envelope (E) proteins (vIND-ZIKVs). These vIND-ZIKVs contain elements of the *tetracycline* operon and replicate only in the presence of tetracyclines. The pool of vaccine candidates was narrowed to one vIND-ZIKV containing a novel mutation in the signal peptide of prM that led to higher expression and secretion of E and production of virus-like particles, which was then tested for safety, immunogenicity, and efficacy in mice. vIND-ZIKV grows to high titers in vitro in the presence of doxycycline (DOX) but is replication-defective in vivo in the absence of DOX, causing no weight loss in mice. C57BL/6 mice vaccinated once with vIND-ZIKV in the absence of DOX (as a replication-defective virus) developed robust levels of E-peptide-specific IFN-γ-secreting splenocytes and anti-E IgG titers, with modest levels of serum-neutralizing antibodies. Vaccinated mice treated with anti-IFNAR1 antibody were completely protected from ZIKV viremia post-challenge after a single dose of vIND-ZIKV. Furthermore, mice with prior immunity to VACV developed moderate anti-E IgG titers that increased after booster vaccination, and were protected from viremia only after two vaccinations with vIND-ZIKV.

## Introduction

Zika virus (ZIKV) is a member of the *Flaviviridae* family, a group of viruses that contain a positive-sense ssRNA genome about 11 kb in length. The ZIKV genome encodes a single polyprotein which is cleaved by viral and cellular proteases into three structural proteins (capsid, C; pre-membrane, prM; and envelope, E) and several non-structural (NS) proteins^1,2^. ZIKV is primarily transmitted by bites of infected *Aedes* mosquitos, but can also be transmitted from mother to fetus, or through sexual contact, breastfeeding, or blood transfusion^3^.

ZIKV was first isolated from a sentinel monkey in the Zika forest of Uganda in 1947^4^. The first human case was reported in 1960 in Nigeria, followed by limited sporadic cases until the 2007 outbreak on Yap Island in Micronesia, during which an estimated 73% of the residents became infected with ZIKV^5^. A major epidemic of ZIKV infection occurred in French Polynesia in 2013–2014 with an estimated 19,000 suspected cases of ZIKV^6^. In May 2015, authorities in Brazil confirmed autochthonous transmission of ZIKV and within 5 months, it had spread to 14 states within Brazil^7^. In late 2015, increasing numbers of infants born with microcephaly were reported, prompting the Brazil Ministry of Health to declare a Public Health Emergency of National Importance^8^ and the World Health Organization to declare a Public Health Emergency of International Concern from February-November 2016^9^. Once it emerged in Brazil, ZIKV spread rapidly throughout Central and South America, leading to over 170,000 confirmed ZIKV cases across 48 countries and territories^3^.

The rapid spread of ZIKV and its association with neurological diseases necessitated the rapid development of a safe and efficacious vaccine. Since the 2015 outbreak, there has been considerable effort to develop vaccines against ZIKV. Vaccine candidates to date are based on several different platforms, including purified inactivated virus, live-attenuated viruses, DNA, mRNA, protein, peptide, and viral-vectored vaccines^10^. Most flavivirus vaccine candidates are based on the E protein (since E is the target of neutralizing antibodies^1^) or co-expression of prM and E, to lead to the formation of virus-like particles (VLPs)^11^.

Vaccinia virus (VACV) was used to eradicate smallpox, a disease caused by variola virus, a related poxvirus. VACV has also been used as a viral vector for the development of effective human and animal vaccines since it is thermally stable, able to elicit strong humoral and cell-mediated immune (CMI) responses, easy to propagate, and not oncogenic^12^. However, VACV can cause complications in individuals with conditions such as atopic dermatitis, cardiac disease, and immunosuppression. We recently generated VACV vectors with a built-in safety mechanism that replicate only in the presence of tetracycline antibiotics^13,14^. The replication-inducible VACVs (vINDs) contain elements from the *tetracycline* (*tet*) operon, specifically the *tetR* gene encoding the repressor protein (TetR), along with the *tetO*_*2*_ operator sequence downstream of the promoter of a gene essential for VACV replication (e.g., D6R, A7L, A6L)^13,14^. In the absence of tetracyclines, the TetR protein is expressed and binds to the operator sequence, preventing transcription of the essential gene, and consequently replication of the virus. Conversely, in the presence of tetracyclines such as doxycycline (DOX), the TetR protein undergoes a conformational change and no longer binds the operator sequence, allowing transcription of the essential gene and replication of the virus. In the absence of antibiotics, vINDs do not produce infectious progeny^13,14^ and act like other replication-deficient VACV strains such as modified vaccinia Ankara (MVA). Unlike MVA, vINDs replicate to high titers in standard cell culture in the presence of tetracycline antibiotics. Importantly, in the absence of inducer, expression of a fluorescence marker is detected in abortively-infected cells^13,14^, indicating that even in the absence of viral replication, heterologous antigens are expressed.

Here, we generated several ZIKV vaccine candidates in a vIND backbone (as a viral vector) to express ZIKV as secreted VLPs, in an effort to induce robust ZIKV immunity by both the viral vector and by the secreted VLPs. We found that a novel mutation (D4W) in the natural signal peptide (SP) of prM resulted in increased expression and secretion of E. We chose this vIND-ZIKV to continue into downstream studies. A single dose of vIND-ZIKV induced robust CMI and humoral immune responses that protected transiently-susceptible C57BL/6 mice from viremia after ZIKV challenge. We also tested our vaccine in the context of prior VACV immunity and found that mice previously inoculated with vIND required two doses of vIND-ZIKV to generate high anti-E antibody titers and protect against ZIKV viremia.

## Results

### Design and generation of ZIKV vaccine candidates

**Figure 1:**
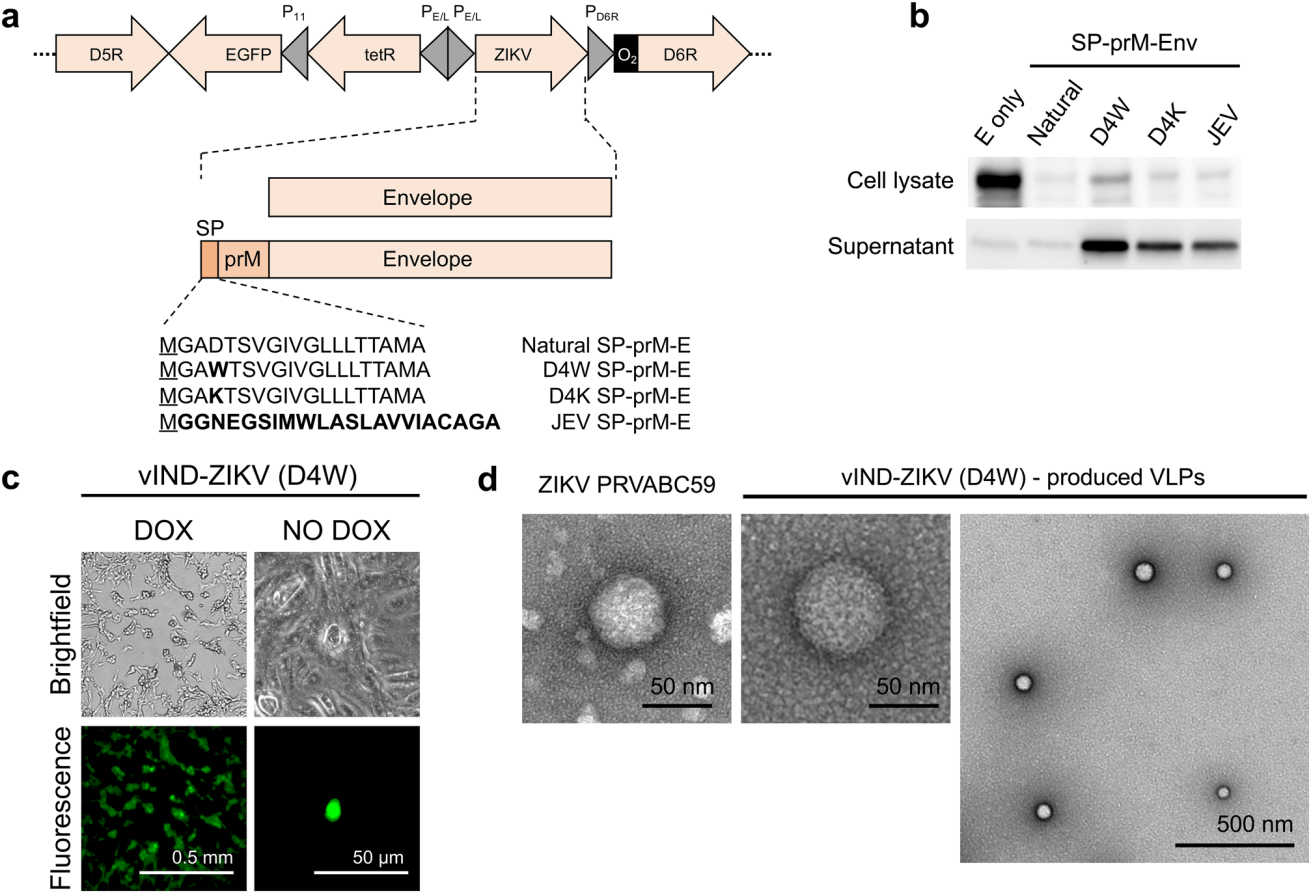
vIND-ZIKV encoding prM and E secretes E into the supernatant of infected cells and forms VLPs. (**a**) Schematic representation of vIND-ZIKV vaccine constructs in the D5R-D6R locus of VACV. Constructs contained the *tet* repressor gene (*tetR*) under the control of a strong synthetic early/late promoter (P_E/L_), the *tet* operator sequence (O_2_) immediately downstream of the natural D6R promoter (P_D6R_), and the EGFP gene under the control of the natural F17R late promoter (P_11_). Signal peptide (SP) variant sequences are listed; underlined M represents methionine added to N-terminus and bold amino acids represent mutations from the natural SP sequence or the JEV SP sequence. (**b**) Western blot of Vero cells (lysates or supernatants) infected with the vaccine candidates. Bands of approximately 55 kDa were observed. Full-length blots are presented in Supplementary Figure 3. (**c**) Representative brightfield and fluorescence images of cells 2 days after infection with vIND-ZIKV (D4W), showing plaque formation in the presence of DOX (cytopathic effects on multiple EGFP^+^ cells), or abortive infection (single EGFP^+^ cell) in absence of DOX. (**d**) Representative TEM images of ZIKV PRVABC59 virion (left) or VLPs secreted into the supernatant of vIND-ZIKV (D4W)-infected Vero cells.

We generated several vaccine candidates against ZIKV based on a sequenced isolate (Asian lineage), Brazil-ZKV2015 (accession #KU497555.1)^15^. A schematic representation of the vaccine constructs is shown in Fig. 1a. The ZIKV gene(s) were placed under the control of a synthetic VACV P_E/L_ promoter^16^ and inserted between VACV genes D5R and D6R by homologous recombination, generating a vIND that replicates only in the presence of tetracyclines^13^. Enhanced green fluorescence protein (EGFP) was included in the recombinant VACVs (rVACVs) to expedite purification. The first vaccine candidate contained the full-length E protein with a methionine added immediately upstream to facilitate translation. A second vaccine candidate was designed that included prM and E, along with the putative natural signal peptide (SP) encoded in the last 18 amino acids of C^2^, to ensure proper folding and secretion of E and lead to the formation of VLPs^11^ (Fig. 1a).

SPs characteristically contain three distinct domains: an N-terminal (n) region often containing positively-charged residues, a hydrophobic (h) region of at least six hydrophobic residues, and a polar uncharged C-terminal (c) region^17^ to facilitate translocation into the endoplasmic reticulum (ER) and in the case of ZIKV capsid SP, to direct prM into the ER lumen for proper secretion of E. Upon reviewing the ZIKV SP sequence, we were concerned that the negatively-charged aspartic acid in the n-region (Fig. 1a) would lead to sub-optimal secretion of E. We designed a series of SP variants using TargetP 1.1 software^17^ to evaluate the localization of proteins based on the SP sequence. The first variant we generated replaced the aspartic acid residue with a strongly hydrophobic residue, of which tryptophan resulted in the highest secretory pathway prediction score (0.930), compared to the natural SP (score 0.865). We also generated a SP variant that replaced the aspartic acid with a positively charged lysine (score 0.878). Lastly, we generated a vaccine candidate that included the last 22 amino acids of the SP of Japanese Encephalitis Virus (JEV, score 0.931), since this sequence has been used successfully to target proteins for secretion^18^.

Once constructs containing the desired ZIKV antigens were generated (Fig. 1a), they were subcloned into a plasmid containing elements of the tetracycline (*tet*) operon^13^ to facilitate generation of vINDs expressing the ZIKV antigens (vIND-ZIKVs). The resulting shuttle vectors were transfected into cells infected with a *lac* operon-based inducible parental virus, and after homologous recombination, vIND-ZIKVs were purified away from parental virus using our recently developed accelerated method^19^. Briefly, cells were serially infected with the parental VACV/rVACV pool in the presence of DOX (rVACV inducer) and absence of isopropyl β-d-1-thiogalactopyranoside (IPTG, parental VACV inducer). Using this method, single clones of each vIND-ZIKV were obtained in about 1 week.

### Single mutations within the SP of prM result in increased secretion of E

The five vaccine candidates (Fig. 1a) were then evaluated for expression of E in both the lysate and supernatant of Vero cells infected in the absence of DOX by western blot (Fig. 1b). A protein matching the expected size of E (~ 55 kDa) was observed in the cell lysate for all vaccine constructs, albeit to different levels. As expected, in cells infected with the vIND-ZIKV expressing E only (without SP and prM) expression of E was contained within the cell (cell lysate), with little secretion of E detected in the supernatant (Fig. 1b). Interestingly, similarly low levels of E were secreted into the supernatant by the natural SP, but those levels increased dramatically when the natural SP was replaced with each of the three variants, especially D4W. Similar results were observed when Vero cells were infected with the vIND-ZIKVs in the presence of DOX (Supplementary Figure 1). This increase in expression and secretion of E by vIND-ZIKV (D4W) was also detected in human HeLa S3 cells, which poorly secreted E (Supplementary Figure 2). Therefore, substitution of the D residue with W dramatically increased expression of E in the cell lysate and supernatant compared to the natural SP, and resulted in the strongest expression of E compared to all variants tested. Thus, vIND-ZIKV (D4W) was selected as the vaccine candidate to progress to further studies (referred to from now on as vIND-ZIKV).

### vIND-ZIKV produces ZIKV VLPs

Replication of vIND-ZIKV in the absence and presence of DOX was evaluated by fluorescence microscopy. As expected, formation of plaques (EGFP^+^) was observed only in the presence of DOX, whereas single abortively-infected cells were detected by expression of EGFP in the absence of DOX (Fig. 1c). vIND-ZIKV was also evaluated for formation of VLPs by transmission electron microscopy (TEM) compared to wild-type ZIKV particles. Stock ZIKV strain PRVABC59 and supernatants from vIND-ZIKV-infected cells were concentrated and negatively stained with 0.5% uranyl acetate for TEM imaging. We visualized VLPs of the expected size (~ 50–60 nm^20^) in the supernatant of vIND-ZIKV-infected cells that resembled virions produced by ZIKV PRVABC59 (Fig. 1d).

### vIND-ZIKV grows to high titers in the presence of DOX but does not replicate in the absence of DOX

**Figure 2:**
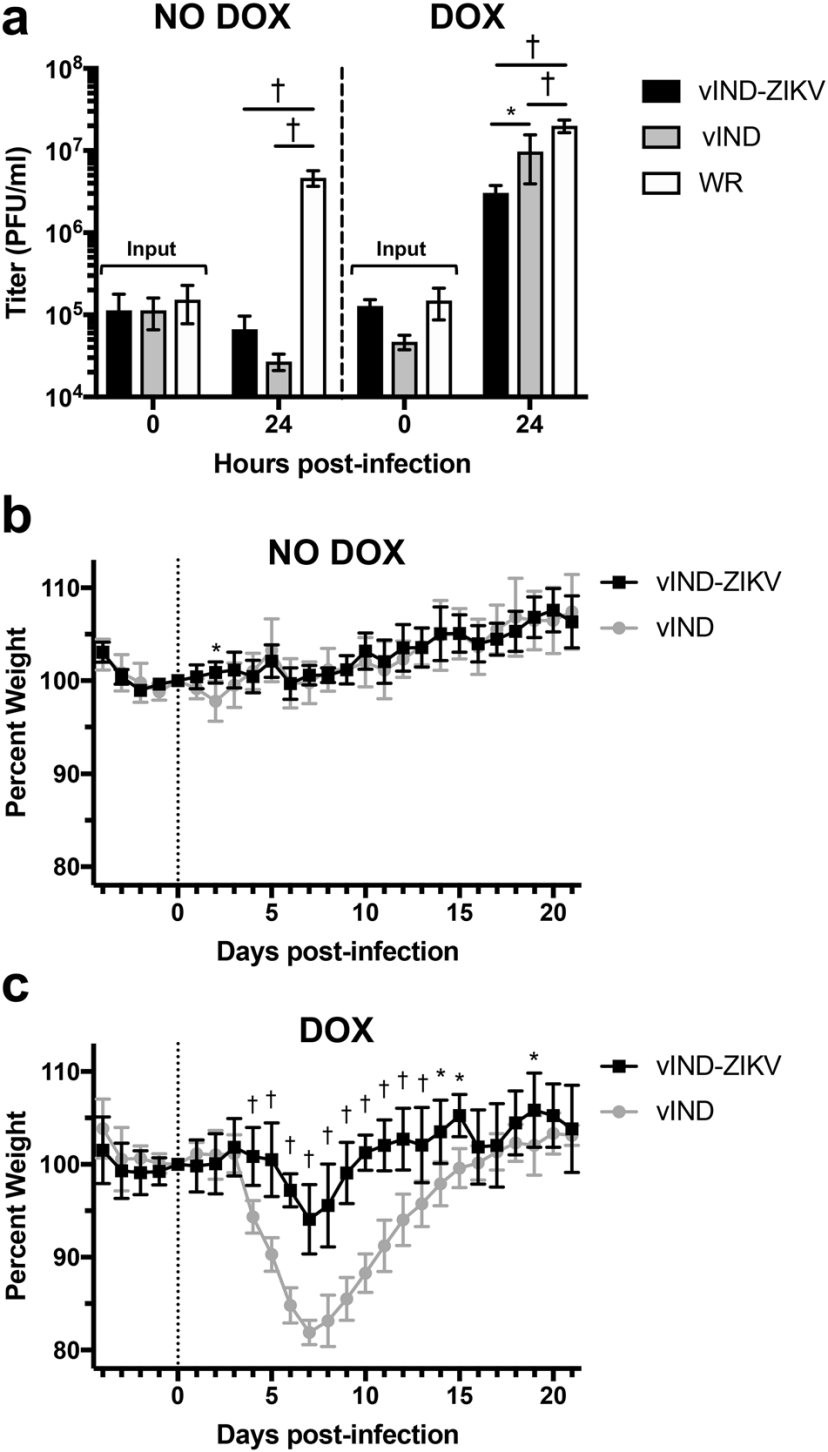
vIND-ZIKV replicates only in the presence of DOX and is attenuated compared to vIND in vitro and in vivo. (**a**) BS-C-1 cells were infected with wild-type strain Western Reserve (WR), vIND or vIND-ZIKV (D4W) at an MOI of 5 in the absence or presence of 1 µg/ml DOX. At 0 or 24 h, cells were collected and lysates were titered in duplicate on BS-C-1 cells in the presence of 1 µg/ml DOX. Plaques were counted 2 days post-infection and mean titers of triplicate samples are shown. (**b**,**c**) CB6F_1_ mice (n = 5) were inoculated intranasally with 2 × 10^4^ PFU vIND or vIND-ZIKV (D4W) in either the absence (**b**) or presence (**c**) of 0.125 mg/ml DOX in the drinking water and were weighed daily for 21 days. Asterisks represent statistical significance (*p < 0.05, ^†^p < 0.001) by two-way ANOVA (**a**) or two-way repeated measures ANOVA (**b**,**c**). Error bars represent SD.

Next, the replication of vIND-ZIKV was evaluated in vitro (Fig. 2a). BS-C-1 cells were infected with vIND-ZIKV, vIND, or the wild-type (replication-competent) strain Western Reserve (WR) at a multiplicity of infection (MOI) of 5 in the absence or presence of 1 µg/ml doxycycline (DOX). Cells were collected at 0 and 24 h post infection (hpi) and lysates were titered on fresh monolayers in the presence of DOX. In the absence of DOX, vIND-ZIKV and vIND did not replicate (titers at 24 hpi were lower than input levels at 0 hpi), while the replication-competent WR replicated to high titers. In the presence of DOX, vIND reached near-wildtype levels of replication by 24 hpi, albeit statistically significantly lower than WR; however, vIND-ZIKV replication was attenuated compared to both WR and vIND (p < 0.001 and p < 0.05, respectively). Despite the attenuation of vIND-ZIKV in vitro, high titer vaccine stocks were still readily generated in the presence of DOX for downstream studies.

### vIND-ZIKV is attenuated in mice even in the presence of DOX

To evaluate the safety of vIND-ZIKV, 6-week-old CB6F_1_ mice were inoculated intranasally with 2 × 10^4^ PFU vIND-ZIKV or vIND in either the absence or presence of DOX in the drinking water and were weighed daily for 21 days (Fig. 2b,c, respectively). Intranasal infection of normal mice is an ideal route for studies of poxvirus pathogenesis and virulence, since replication-competent VACVs lead to infection of the central nervous system and weight loss^21^, and this dose was shown to cause weight loss without mortality in vIND-infected mice during pilot studies. Our vINDs are replication-defective in the absence of DOX and should therefore be safer, yet they cause weight loss and mortality (intranasal inoculation) and replicate to wild-type levels in ovaries (intraperitoneal inoculation) in the presence of DOX (as replication-competent VACVs)^14^. Accordingly, in the absence of DOX, mice in both groups maintained and then gained weight throughout the study (Fig. 2b). In a recent study we have shown that vIND replication is not detected in ovaries of mice inoculated intraperitoneally in the absence of DOX^14^. In the presence of DOX, vIND-infected mice started to lose weight on day 4, reached peak weight loss at day 7, and recovered back to starting weight by day 16 (Fig. 2c). However, vIND-ZIKV was slightly more attenuated than vIND in the presence of DOX, as mice infected with vIND-ZIKV lost weight to a lesser degree than those infected with vIND (p < 0.001). This demonstrated that our vIND-ZIKV vaccine would be safe when given as a vaccine in the absence of DOX (as a replication-defective vaccine vector) and has an added safety feature, since it is attenuated (compared to vIND) even in the presence of DOX.

### vIND-ZIKV induces high levels of CMI responses in mice

**Figure 3:**
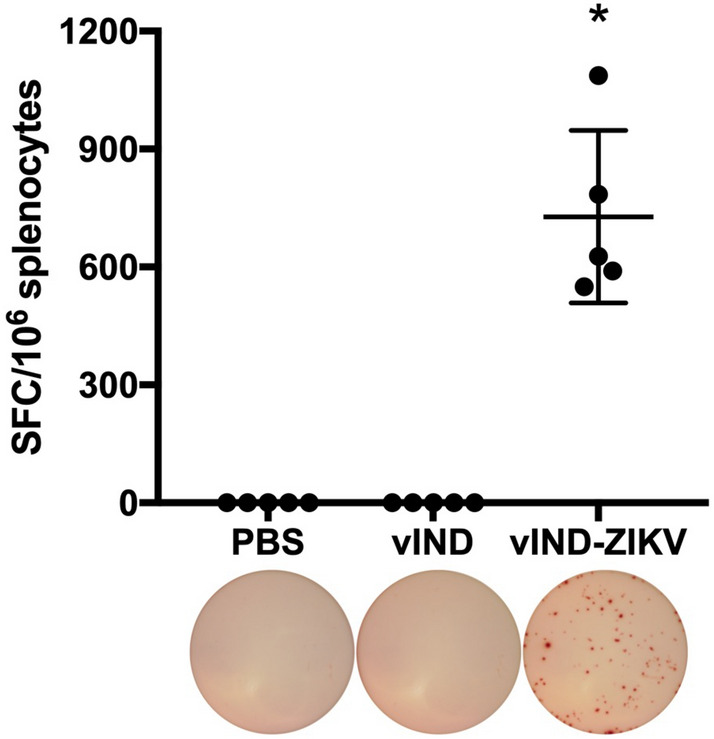
vIND-ZIKV vaccination stimulates antigen-specific IFN-γ-secreting splenocytes after 1 week. Antigen-specific IFN-γ-secreting splenocytes in C57BL/6 mice (n = 5) vaccinated intramuscularly with either PBS, vIND, or vIND-ZIKV (D4W) at 10^7^ PFU were measured 7 days post vaccination by ELISPOT with E peptide IGVSNRDFVEGMSGG. Data is shown as spot-forming cells (SFC) per 10^6^ splenocytes. Asterisk represents statistical significance (p < 0.01) with the Kolmogorov–Smirnov test when comparing to the PBS-vaccinated control group. Horizontal line represents mean and error bars represent SD. Images of representative wells are shown below each group.

We next sought to evaluate the immunogenicity of our vaccine candidate. We first tested CMI by vaccinating 6-week-old C57BL/6 mice (n = 5) intramuscularly with 10^7^ PFU vIND, vIND-ZIKV, or PBS (in the absence of DOX). After 7 days, mice were sacrificed, spleens were removed, and splenocytes were harvested and incubated with 4 µg/ml of a 15-mer peptide of ZIKV E protein for 18 h for an ELISPOT assay. Mice vaccinated with vIND-ZIKV had robust levels of antigen-specific IFN-γ-secreting splenocytes that were not detected in mice vaccinated with PBS (p < 0.01) or vIND (Fig. 3).

### vIND-ZIKV induces humoral immune responses in mice

**Figure 4:**
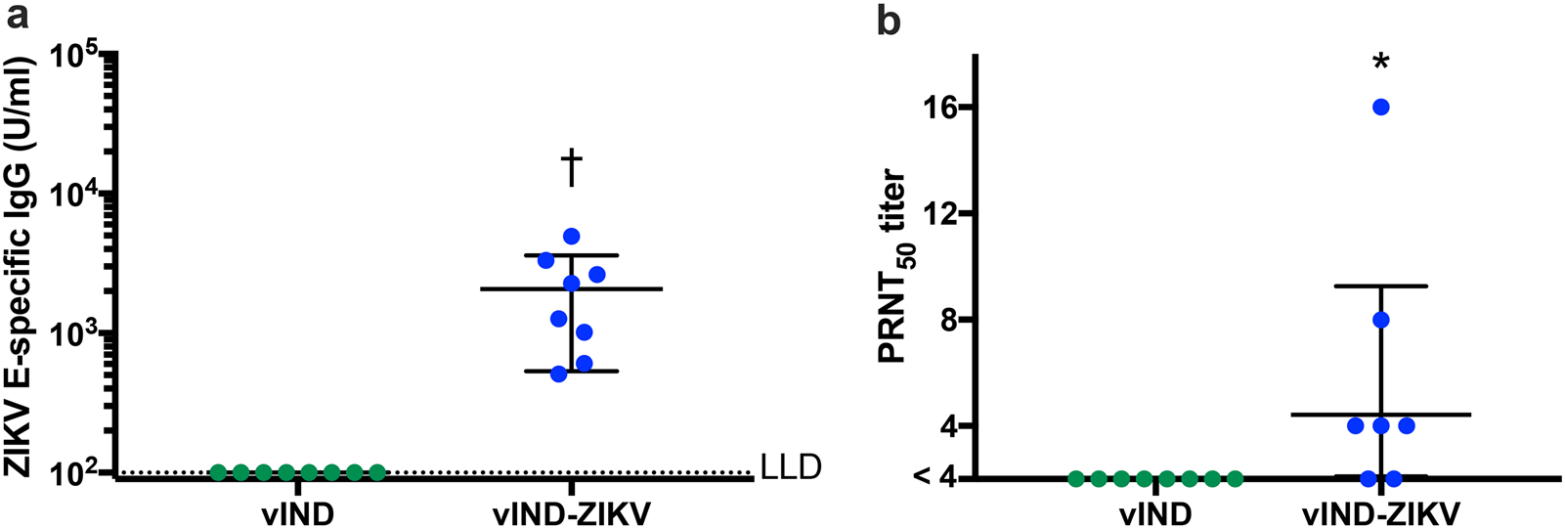
vIND-ZIKV vaccination induces E-specific IgG and neutralizing antibodies. Humoral immune responses in C57BL/6 mice (n = 8) vaccinated intramuscularly with 10^7^ PFU vIND or vIND-ZIKV (D4W) are shown 4 weeks after vaccination. (**a**) ZIKV E-specific IgG titers were measured by ELISA at week 4. (**b**) Plaque reduction neutralization tests (PRNTs) were performed on serum collected from mice vaccinated with vIND or vIND-ZIKV. The PRNT_50_ was calculated as the reciprocal of the dilution that resulted in at least 50% reduction in ZIKV plaques. Naïve sera (week 0) had PRNT_50_ titers < 4 (data not shown). Asterisk represents statistical significance (*p < 0.01, ^†^p < 0.001) with the Kolmogorov–Smirnov test. Horizontal lines represent geometric mean and error bars represent SD. *LLD* lower limit of detection.

We analyzed the humoral immune responses of vIND-ZIKV by measuring the induction of ZIKV E-specific IgG and neutralizing antibodies (Fig. 4). Six-week-old C57BL/6 mice (n = 8) were vaccinated intramuscularly with 10^7^ PFU vIND or vIND-ZIKV. Blood was collected on the day of vaccination (naïve sera) or at euthanasia (4 weeks after vaccination) for analysis. Antibodies against ZIKV E were measured by ELISA (Fig. 4a). vIND-vaccinated mice had no E-specific IgG titers after vaccination, while vIND-ZIKV vaccination induced robust levels of E-specific antibodies (geometric mean 2072 U/ml, p < 0.001). Next, neutralizing antibodies in serum were measured by plaque reduction neutralization test (PRNT) (Fig. 4b). As expected, serum from vIND-vaccinated mice did not neutralize ZIKV (PRNT_50_ titer < 4). Surprisingly, mice inoculated with vIND-ZIKV had low neutralizing antibody titers (geometric mean PRNT_50_ titer of 4.4) after vaccination, although they were statistically higher than vIND (p < 0.01). One vIND-ZIKV-vaccinated mouse was excluded from analysis due to low volume of serum collected that prevented analysis at the lowest dilution, although this mouse had a PRNT_50_ titer < 6. Despite low neutralizing antibody titers, vIND-ZIKV vaccination induced robust levels of E-specific IgG (Fig. 4a) and antigen-specific CMI (Fig. 3) that warranted further investigation in a challenge model.

### vIND-ZIKV induces humoral immune responses and protects mice from viremia

**Figure 5:**
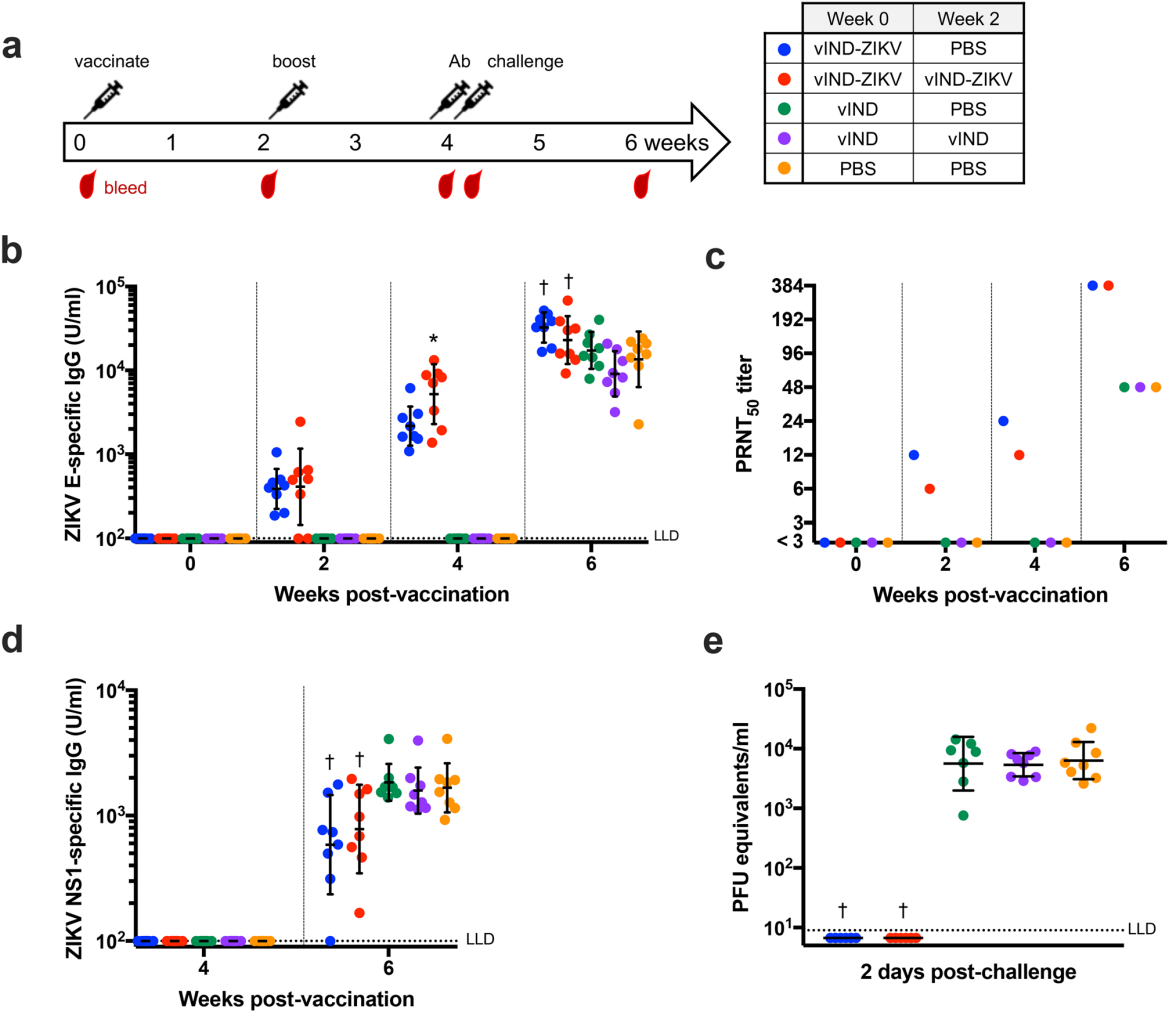
A single vaccination with vIND-ZIKV is protective in mice. (**a**) Schematic of vaccination/challenge schedule and timing of blood collection. Six-week-old immunocompetent C57BL/6 mice (n = 8) were vaccinated intramuscularly with PBS or 10^7^ PFU vIND or vIND-ZIKV (D4W) at weeks 0 and 2. Mice were challenged 2 weeks post-boost with 10^4^ PFU ZIKV strain PRVABC59 intraperitoneally, 1 day after being administered 2 mg anti-IFNAR1 antibody intraperitoneally. (**b**) ZIKV E-specific IgG titers were measured by ELISA at weeks 0, 2, 4 (1 day prior to challenge), and 6. (**c**) PRNTs were performed in twofold dilutions on pooled serum collected at the indicated time points. (**d**) ZIKV NS1-specific IgG titers were measured by ELISA at weeks 4 (1 day prior to challenge) and 6. (**e**) Viremia was measured in serum collected 2 days after ZIKV challenge by qRT-PCR. Asterisks represent statistical significance (*p < 0.05, ^†^p < 0.005) by two-way repeated measures ANOVA (**b**,**d**) or unpaired t tests (**e**) compared to PBS-vaccinated control group. Horizonal lines represent geometric mean and error bars represent SD. *LLD* lower limit of detection.

To further assess the humoral immune responses and simultaneously evaluate efficacy of vIND-ZIKV, we utilized a ZIKV challenge model^22^ where C57BL/6 mice are made transiently susceptible to ZIKV infection by administering an anti-IFNAR1 monoclonal antibody^23^ 1 day prior to challenge. Since low neutralizing antibody titers were observed after a single vIND-ZIKV vaccination (Fig. 4b), a group receiving two vIND-ZIKV vaccinations was included (as indicated in Fig. 5). Briefly, 6-week-old C57BL/6 mice (n = 8) were vaccinated intramuscularly with PBS or 10^7^ PFU vIND or vIND-ZIKV at weeks 0 and 2, as outlined in Fig. 5a. Mice were challenged 2 weeks post-boost with 10^4^ PFU ZIKV strain PRVABC59 (Asian lineage) intraperitoneally, 1 day after administration of 2 mg anti-IFNAR1 antibody (Leinco, MAR1-5A3) intraperitoneally. Mice were sacrificed 2 weeks later at the conclusion of the study. Blood was collected retro-orbitally at regular intervals (Fig. 5a) or by cardiac puncture at euthanasia.

To measure the humoral immune response to vIND-ZIKV, antibody titers against E were analyzed by ELISA (Fig. 5b). In mice vaccinated once with vIND-ZIKV, E-specific IgG titers were low (geometric mean 387 U/ml) 2 weeks post vaccination, but increased by 4 weeks post vaccination (geometric mean 2166 U/ml). Similarly, mice vaccinated twice with vIND-ZIKV had low anti-E titers following the first vaccination (geometric mean 411 U/ml), but increased tenfold after a second vaccination with vIND-ZIKV (geometric mean 5214 U/ml; p < 0.05 compared to mice inoculated with PBS). Following ZIKV challenge, control groups vaccinated with vIND (once or twice) or PBS developed anti-E IgG titers (geometric mean of 17,291; 9078; or 13,578 U/ml, respectively). E-specific antibody titers were boosted post-challenge in mice vaccinated once or twice with vIND-ZIKV (geometric mean 32,509 or 22,964 U/ml, respectively), and were statistically significantly higher than mice inoculated with PBS (geometric mean 13,578 U/ml; p < 0.005) (Fig. 5b).

The level of neutralizing antibodies was determined for each group at weeks 0, 2, 4, and 6. Since only small volumes of blood were collected retro-orbitally at each time point, serum from each group was pooled for PRNT analysis. Mice vaccinated with vIND-ZIKV had a modest increase in neutralizing titer at weeks 2 and 4 followed by an increase after challenge (Fig. 5c). Next, to assess the extent to which vIND-ZIKV protected against ZIKV replication after challenge, antibody titers against NS1 were measured by ELISA. Most mice vaccinated with vIND-ZIKV once or twice had detectable NS1 titers at week 6 (2 weeks post-challenge), although significantly lower when compared to PBS-vaccinated controls (p < 0.005) (Fig. 5d), indicating potential challenge virus replication (i.e., lack of sterilizing immunity).

C57BL/6 mice made transiently susceptible to ZIKV by anti-IFNAR1 antibody develop viremia after challenge^22^. We collected blood 2 days post-challenge (determined in pilot studies as the day of peak viremia), and performed quantitative reverse transcription PCR (qRT-PCR) using primers previously described^24^. Mice vaccinated with PBS or vIND once or twice had high levels of viremia (Fig. 5e). However, mice vaccinated once or twice with vIND-ZIKV were protected from viremia (p < 0.005). Therefore, a single dose of vIND-ZIKV was sufficient to completely protect mice from transient viremia.

### VACV-primed mice vaccinated twice with vIND-ZIKV are protected from viremia

**Figure 6:**
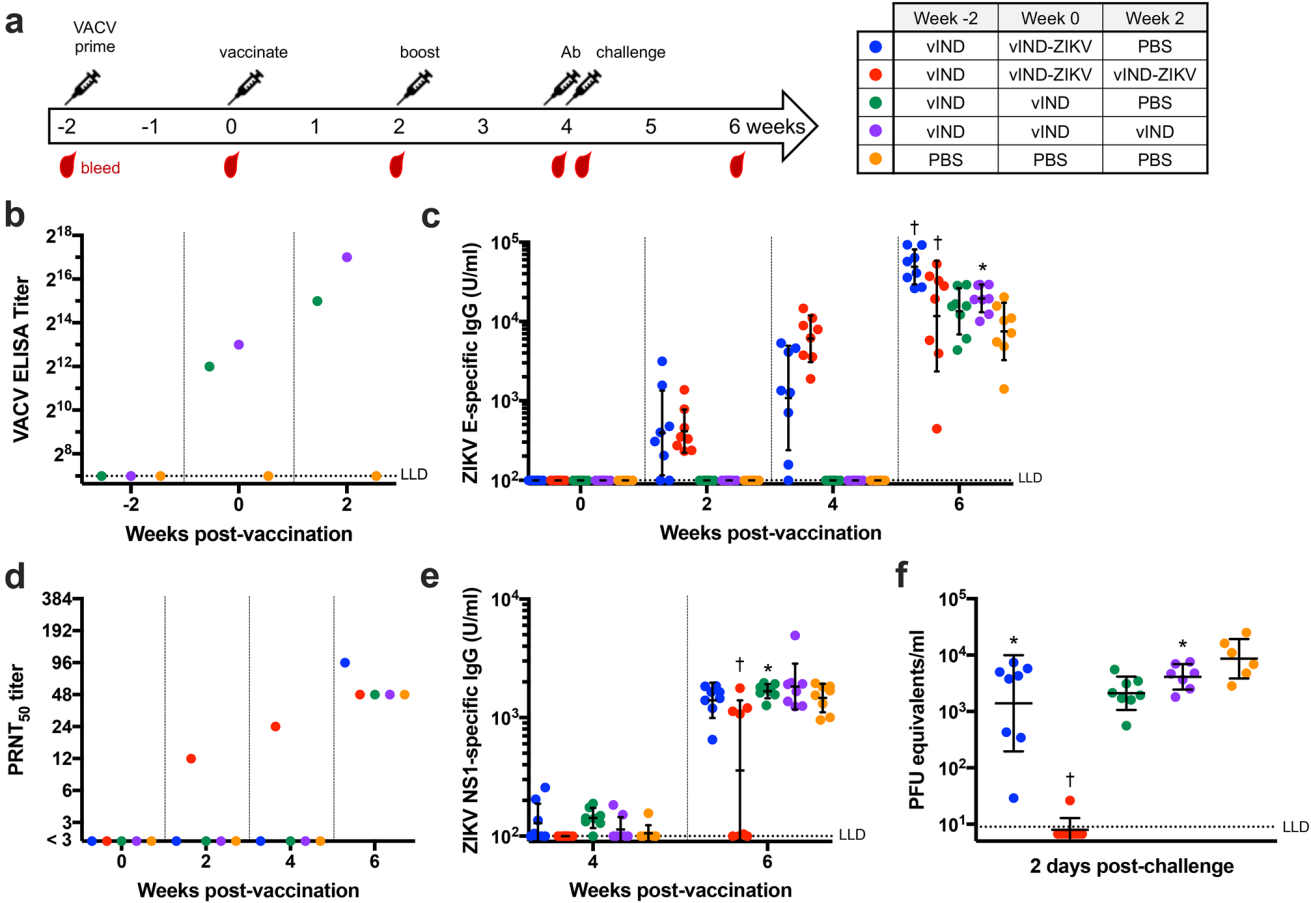
Two vaccinations with vIND-ZIKV are required to protect mice against ZIKV in mice with prior vector immunity. (**a**) Schematic of vaccination/challenge schedule and timing of blood collection in VACV-primed mice. Six-week-old immunocompetent C57BL/6 mice (n = 8) were vaccinated intramuscularly with PBS or 10^7^ PFU vIND 2 weeks prior to first vaccination. Mice were then vaccinated with PBS or 10^7^ PFU vIND or vIND-ZIKV (D4W) at weeks 0 and 2. Mice were challenged 2 weeks post-boost with 10^4^ PFU ZIKV strain PRVABC59 intraperitoneally, 1 day after being administered 2 mg anti-IFNAR1 antibody intraperitoneally. (**b**) Anti-vector (VACV) antibodies were measured by ELISA in pooled serum collected at weeks − 2, 0, and 2. (**c**) ZIKV E-specific IgG titers were measured by ELISA at weeks 0, 2, 4 (1 day prior to challenge), and 6. (**d**) PRNTs were performed in twofold dilutions on pooled serum collected at the indicated time points. (**e**) ZIKV NS1-specific IgG titers were measured by ELISA at weeks 4 (1 day prior to challenge) and 6. (**f**) Viremia was measured in serum collected 2 days after ZIKV challenge by qRT-PCR. Asterisks represent statistical significance (*p < 0.05, ^†^p < 0.005) by two-way repeated measures ANOVA (**c**,**e**) or unpaired t tests (**f**) compared to PBS-vaccinated control group. Horizonal lines represent geometric mean and error bars represent SD. *LLD* lower limit of detection.

Since VACV can be, and has been, used for many applications (e.g., vaccine, therapeutic, and oncolytic vectors), we also wanted to explore if prior immunity to VACV had any impact on the immunogenicity and efficacy of our ZIKV vaccine. To test this, we repeated the vaccination/challenge experiment described above but added a “VACV prime” 2 weeks prior to vaccination by inoculating mice intramuscularly with 10^7^ vIND (or PBS), in the absence of DOX, to mirror prior vector immunity (Fig. 6a). Anti-VACV antibodies were detected in vIND-primed mice at the time of vaccination (week 0) and were further increased by week 2 (Fig. 6b).

As seen previously in naïve mice (Fig. 5b), mice vaccinated once with vIND-ZIKV had low E-specific IgG titers 2 weeks post vaccination (geometric mean 394 U/ml), but titers increased by 4 weeks post vaccination (mean 1084 U/ml) (Fig. 6c). Mice vaccinated twice with vIND-ZIKV had similarly low anti-E titers 2 weeks after initial vaccination (geometric mean 413 U/ml), which increased nearly 15-fold after the booster vaccination (geometric mean 6050 U/ml). Control groups vaccinated once or twice with vIND, or with PBS only developed anti-E IgG titers after ZIKV challenge (geometric mean of 13,456; 19,521; or 7477 U/ml; respectively). Antibody titers against E increased after ZIKV challenge in mice vaccinated once or twice with vIND-ZIKV (geometric mean 48,884 or 11,719 U/ml, respectively), and were statistically significantly higher than mice vaccinated with PBS (p < 0.005) (Fig. 6c). As seen in the previous challenge study, mice vaccinated with PBS or vIND developed neutralizing antibody titers only after challenge (Fig. 6d). Interestingly, only mice vaccinated twice with vIND-ZIKV had detectable neutralizing antibody titers in pooled sera at weeks 2 and 4, which increased after challenge (Fig. 6d). Similarly, mice vaccinated twice with vIND-ZIKV had reduced NS1-specific antibody titers 2 weeks post-challenge (Fig. 6e).

As above, we collected blood 2 days post-challenge and performed qRT-PCR to measure ZIKV viremia. As expected, VACV-primed mice vaccinated with PBS or vIND had high levels of viremia (Fig. 6f). Interestingly, VACV-primed mice vaccinated once with vIND-ZIKV had high levels of viremia, showing that prior inoculation with vIND in the absence of DOX most likely resulted in vector immunity that interfered with vIND-ZIKV vaccination. This finding corresponded with the lack of detectable ZIKV neutralizing antibody titer in this group (Fig. 6d). However, a second vaccination with vIND-ZIKV protected VACV-primed mice from viremia, as levels were statistically significantly lower than the PBS control group (p < 0.005), and were close to or below the detection limit (Fig. 6f).

## Discussion

We developed several vaccine candidates expressing ZIKV E protein either alone or with prM to rapidly determine an antigen strategy that leads to high levels of E protein expression and VLP secretion. Our vaccine candidates were generated in the backbone of a vIND^13^ developed to improve the safety profile of a traditional replication-competent VACV, while still achieving high titers in vitro in traditional cell lines, unlike traditional replication-defective VACVs such as MVA, that do not replicate optimally in standard mammalian cell lines. When administered as a vaccine (in the absence of DOX), our vINDs still express all viral antigens in infected cells and are therefore immunogenic, yet safer for both the vaccine administrator and recipient. In addition, recombinant vINDs can be produced in a week, using our Efficient Purification by Parental Inducer Constraint (EPPIC) system^19^, thus allowing the straightforward generation of multiple vaccine candidates that can be tested for expression, and if needed, immunogenicity.

Here we showed that single mutations in the SP of prM resulted in increased expression of E intracellularly and in the supernatant of vIND-ZIKV-infected cells, compared to the putative natural SP. Since we hypothesized that increased expression and secretion of E would lead to improved immunogenicity of our vaccine, we selected the vaccine candidate with a D4W mutation in the SP to progress to further studies. We also showed that, even in the absence of DOX, co-expression of prM and E (but not E alone) by vIND-ZIKV leads to the formation of VLPs that can be visualized by TEM and resemble wildtype ZIKV virions. VLPs are an appealing vaccine approach because they present epitopes in a conformation similar to the native virus and therefore induce strong innate and adaptive immune responses, are safe because they lack a viral genome and are non-replicating, and contain a highly repetitive surface that is highly immunogenic^25^. Thus, VLPs have been studied as vaccines against ZIKV by several groups^26–34^. In the future, we plan to generate and purify vIND-ZIKV-produced VLPs and vaccinate mice with the purified VLPs directly.

Though our vaccine would be administered only in the absence of DOX, we evaluated growth characteristics in vitro and in vivo both in the absence and presence of DOX. We found that in the absence of DOX, vIND and vIND-ZIKV were replication-defective, demonstrated by input-levels of virus 24 hpi and lack of weight loss in mice. In the presence of DOX, vIND and vIND-ZIKV are replication-competent, reaching high titers in vitro and causing weight loss in mice. Interestingly, vIND-ZIKV was attenuated both in vitro and in vivo compared to vIND. This is not entirely unexpected, since ZIKV VLPs produced by vIND-ZIKV would compete with VACV for cellular resources and processes that may interfere with vIND-ZIKV replication. Regardless, vIND-ZIKV would always be administered in the absence of DOX (and therefore be replication-defective) when given as a vaccine, and is still able to grow to high titers in cell culture during vaccine production (in the presence of DOX).

Here we demonstrated that mice vaccinated with a single dose of vIND-ZIKV had robust levels of ZIKV E-specific IFN-γ-secreting splenocytes 1 week after vaccination. Furthermore, C57BL/6 mice vaccinated with vIND-ZIKV had robust E-specific antibody titers, although PRNT_50_ titers were only modest after a single vaccination. Our PRNT assay measures ZIKV neutralization by reduction in the actual number of plaques developed, which may have resulted in low sensitivity compared to other methods. Importantly, despite low neutralizing antibody titers, vIND-ZIKV-induced immunity was sufficient to protect against ZIKV challenge. After treatment with an anti-IFNAR1 antibody followed by ZIKV challenge, mice vaccinated with one or two doses of vIND-ZIKV were protected from viremia and had reduced anti-NS1 antibody titers, while control mice had high levels of viremia and NS1-specific antibodies.

Though widespread smallpox vaccination has ceased, there is a population of individuals who will have immunity to VACV from either prior immunization as a child, or from more recent vaccination due to employment as a healthcare professional, first responder, military member, or researcher working with poxviruses. Furthermore, VACV is being explored as a vector for vaccines, immunotherapies, and oncolytic therapies^12^. Therefore, it was important to test whether prior immunity to VACV would interfere with the immunogenicity and efficacy of our vaccine. To test this, we inoculated mice with vIND 2 weeks prior to the start of the immunogenicity and efficacy study. Despite prior vector immunity, vaccinated mice generated E-specific antibodies following vaccination once or twice with vIND-ZIKV. Interestingly, while naïve mice vaccinated once with vIND-ZIKV were protected from viremia, mice with prior VACV exposure followed by a single vaccination with vIND-ZIKV were not protected. However, a second vaccination with vIND-ZIKV overcame this inhibitory effect and mice were protected from viremia.

It is important to note that this prior immunity study was very stringent, since the vaccination regimen was initiated only 2 weeks after VACV priming, when antibody levels are already present and CMI responses to the VACV vector would still be robust. It is very likely that further separation of the VACV prime from the vIND-ZIKV vaccination would lessen the effect of prior VACV immunity on vIND-ZIKV efficacy. Conversely, priming with a replication-competent VACV, rather than vIND, may exacerbate the effect of prior immunity on vIND-ZIKV efficacy. However, robust immune responses are generated against VACV despite replication capacity (e.g., MVA)^35^, so we hypothesized that inoculating with vIND would be sufficient to recapitulate the effect of pre-existing VACV immunity. In fact, we observed that a single inoculation of vIND followed by a single vaccination with vIND-ZIKV resulted in moderate levels of anti-E antibody, yet after challenge NS1-specific titers and viremia were similar to negative controls. Despite this, we showed, even in this stringent scenario, that two vaccinations with vIND-ZIKV can overcome prior immunity to VACV.

In addition to vIND-ZIKV presented here, other replication-defective VACV strains have also been studied as viral-vectored vaccine candidates against ZIKV. An MVA vector expressing ZIKV NS1 induced robust CMI and humoral responses and was protective against lethal intracerebral challenge in immunocompetent mice^36^. Furthermore, an MVA vaccine expressing prM and E elicited strong CMI and humoral responses in normal mice and reduced ZIKV viremia in immunocompromised IFNAR^−/−^ mice after challenge^37^. In addition to MVA, other replication-defective VACV strains have been studied as ZIKV vaccines. For example, a replication-defective VACV-vectored vaccine candidate against chikungunya virus (CHIKV) and ZIKV based on the VACV Copenhagen strain and encoding the structural proteins of CHIKV and prM and E of ZIKV induced ZIKV-neutralizing antibodies in immunocompetent and immunocompromised IFNAR^−/−^ mice and protected IFNAR^−/−^ mice from ZIKV viremia and infection of tissues^38^. Furthermore, vaccination with a DNA plasmid (expressing ZIKV prM and E) followed by a booster vaccination with a non-replicating VACV vector (based on the VACV Tiantan strain and expressing ZIKV E) induced anti-E IgG and neutralizing antibodies in mice^39^.

During this study, we rapidly identified advantageous single mutations within the SP of prM that resulted in increased expression and secretion of ZIKV E protein from a vIND. One possible consequence of the SP mutation is improper folding of E resulting in a diminished immune response. While we did not assess protein folding in this study, we did show that expression of prM-E by vIND-ZIKV results in the secretion of VLPs resembling ZIKV particles, as well as robust CMI and humoral immune responses in mice that protected against ZIKV challenge. The vaccine antigen identified here (D4W SP-prM-E) could be incorporated into other vaccine platforms (e.g., DNA or mRNA vaccines, VLPs alone), particularly in the case of vaccinating pregnant mothers or women of child-bearing age, or could be pursued further in replication-defective vIND vectors with additional safety mechanisms^40^ to counteract the potential loss of TetR protein function due to mutations.

## Materials and methods

### Ethics statement

This study was carried out in accordance with recommendations set forth by the National Institutes of Health Guide for the Care and Use of Laboratory Animals and in compliance with the ARRIVE guidelines. Experimental protocols were approved by the University of Connecticut Institutional Animal Care and Use Committee. All animals were purchased from The Jackson Laboratory (Bar Harbor, ME, USA) and housed in an AAALAC-accredited facility.

### Cells

African green monkey BS-C-1 (CCL-26), Vero (CCL-81), and human HeLa S3 (CCL-2.2) cells were obtained from the American Type Culture Collection (ATCC, Manassas, VA, USA) and were grown in Dulbecco’s modified Eagle medium (D-MEM; Thermo Fisher Scientific, Waltham, MA, USA) supplemented with 5–10% tetracycline-tested fetal bovine serum (FBS, Bio-Techne, Flowery Branch, GA, USA). All cells were grown at 37 °C in 5% CO_2_.

### Viruses, antibodies and peptides

The L-variant of VACV strain Western Reserve (WR) was obtained from ATCC (VR-2035) and a clone (9.2.4.8) derived by sequential plaque purification^40^ was used to generate the recombinant viruses in this study. ZIKV strain PRVABC59 (Asian lineage) was obtained from BEI Resources (National Institute of Allergy and Infectious Disease, National Institutes of Health, USA; NR-50240) and was thawed once and divided into aliquots that were stored at − 80 °C. New aliquots were thawed for each assay and discarded after use. ZIKV envelope protein antibody GTX133314 was obtained from Genetex (Irvine, CA, USA), and goat anti-rabbit IgG secondary antibody PI31460 conjugated to horseradish peroxidase (HRP) was obtained from Thermo Fisher Scientific. Peptides spanning the entire ZIKV envelope protein as consecutive 15-mers with 12-mer overlap were obtained from BEI Resources (NR-50553).

### ZIKV genes

The prM and E genes of ZIKV strain Brazil-ZKV2015 (sequences based on accession #KU497555.1, Asian lineage) were obtained by gene synthesis (Atum, Newark, CA, USA). The entire coding region of E (504 amino acids) was included in the construct with a methionine amino acid at the N-terminus. For constructs containing prM and E, the 18 amino acids preceding prM (the putative signal sequence within the C protein) were encoded immediately upstream of prM (168 amino acids), with a methionine amino acid at the N-terminus. TargetP 1.1 software^17^ (Technical University of Denmark) was used to predict the localization of the E protein (e.g., secretory pathway). Variants of the natural capsid SP were selected based on improvements in the output of TargetP 1.1 software. A 6× His tag was encoded immediately downstream of E in all constructs.

### Construction of vIND-ZIKV transfer plasmids

The ZIKV gene(s) were inserted into a plasmid backbone containing the *tetR* repressor gene under the control of a constitutive VACV promoter and the *tetO*_*2*_ operator sequence, which was inserted directly downstream of the natural D6R promoter to control expression of the VACV gene D6R^13^. To expedite purification of the recombinant viruses, enhanced green fluorescence protein (EGFP) was also included in the construct under the control of a VACV P_11_ (F17R) promoter.

### Generation of vIND-ZIKVs

Recombinant VACVs were generated by infecting BS-C-1 cells in 12-well culture plates with a *lac*-inducible parental virus (viLacR, expressing DsRed fluorescence protein) for 1 h. Infected cells were then overlaid with complete D-MEM supplemented with 2.5% FBS containing 0.1 mM isopropyl β-d-1-thiogalactopyranoside (IPTG) and 1 µg/ml DOX (Sigma-Aldrich, St. Louis, MO, USA). Plasmids were complexed with FuGENE HD transfection reagent (Promega, Madison, WI, USA) for 15 min before being added to individual wells of infected cells. Cells were incubated for 2 days at 37 °C before being analyzed with an EVOS FL inverted fluorescence microscope (Thermo Fisher Scientific) for successful transfection (EGFP expression) and parental virus replication (DsRed expression and cytopathic effect). Cell lysates and supernatants were collected and processed, and vIND-ZIKVs were serially purified from parental virus in the absence of IPTG and presence of DOX by our recently developed method^19^ based on the swapping of inducible systems. High-titer stocks were generated by infecting HeLa S3 cells with the VACVs at an MOI of 0.1 in the presence of 1 µg/ml DOX^14^. The vIND-ZIKVs from high-titer stocks were authenticated by extraction of viral DNA (NucleoSpin Blood Mini kit, Macherey-Nagel, Bethlehem, PA, USA) and PCR amplification with Q5 high-fidelity DNA polymerase (New England Biolabs, Ipswich, MA, USA). The PCR product was checked by restriction enzyme analyses, and either sequenced directly or after cloning into the Zero Blunt PCR cloning kit (Thermo Fisher Scientific). PCR and sequencing primers are shown in Supplementary Table 1.

### Expression of ZIKV proteins from vIND-ZIKVs by western blot

Vero or HeLa S3 cells grown in 100 mm culture dishes to near confluency were infected with each VACV at an MOI of 5. After 1 h, cells were washed and overlaid with D-MEM containing 2.5% FBS with or without the addition of 1 µg/ml DOX, and incubated at 37 °C for 2 days. Cell lysates were collected and processed. Supernatants were clarified by centrifugation (1000  $\times$ g$\times$ for 10 min at 4 °C) and transferred (~ 8 ml) to conical tubes containing 2 ml of ice-cold 40% PEG-8000, and incubated overnight at 4 °C. The 10 ml mixtures were then added to ultraclear centrifuge tubes, loaded onto a SW 32 Ti rotor (Beckman Coulter, Indianapolis, IN, USA), and centrifuged at 9100 rpm for 30 min at 4 °C. Supernatants were discarded and pellets were resuspended in 80 µl 10 mM Tris (pH 8.0) buffer.

Samples were run on 4–20% Mini-PROTEAN TGX Stain-Free gels (BioRad, Hercules, CA, USA) and proteins were then transferred onto mini PVDF membranes using TransBlot Turbo (BioRad). Membranes were incubated in blocking buffer (5% non-fat milk in PBS-Tween) for 1 h, washed with PBS-Tween, and primary antibody was then added and incubated for 2 h. The membranes were then washed 3 times with PBS-Tween before adding secondary antibody and incubating for 1 h. Membranes were washed three times with PBS-Tween, two times with water, prepared for chemiluminescent development by incubation in Clarity Western ECL Substrate (BioRad), and imaged with a ChemiDoc digital imager (BioRad).

The effect of DOX on vIND-ZIKV (D4W) plaque formation was determined by infecting near-confluent BS-C-1 cell monolayers in six-well plates with vIND-ZIKV (D4W) at 50 PFU/well in the absence or presence of 1 μg/ml DOX. Individual plaques and infected cells were imaged 2 days later by brightfield and fluorescence microscopy with an Axio Observer D1 inverted fluorescence microscope (Carl Zeiss, Oberkochen, Germany) using an XF100-2 (EGFP) filter (Omega Optical, Brattleboro, VT, USA).

### Negative staining and electron microscopy

Samples were concentrated in preparation for electron microscopy. A virus stock of ZIKV PRVABC59 was concentrated using an ultra-centrifugal filter unit (MilliporeSigma, Burlington, MA, USA) with a 100 kDa cutoff. To concentrate VLPs produced by vIND-ZIKV (D4W), Vero cells grown in T-175 culture flasks to near confluency were infected at an MOI of 5. After 1 h, cells were washed and overlaid with D-MEM supplemented with 2.5% FBS. After cells were incubated for 2 days at 37 °C, the supernatant was clarified by centrifugation at 500$\times$*g* for 10 min at 4 °C, transferred (~ 24 ml) to a conical tube containing 6 ml cold 40% PEG-8000, and incubated overnight at 4 °C. The supernatant/PEG mixture was loaded onto a SW 32 Ti Rotor (Beckman Coulter) and centrifuged at 9100 rpm for 30 min at 4 °C. The pellet was resuspended in 200 µl 10 mM Tris (pH 8.0) buffer. Concentrated ZIKV and VLPs were fixed in 2% glutaraldehyde for 15 min. Fixed samples (3 µl) were then deposited onto plasma-cleaned carbon-coated copper grids (Electron Microscopy Sciences, Hatfield, PA, USA) and incubated for 2 min. The grids were then washed with 0.5% uranyl acetate and air dried. Grids were imaged with a FEI Tecnai 12 G2 Spirit BioTWIN transmission electron microscope at the University of Connecticut Biosciences Electron Microscopy Laboratory.

### One-step growth VACV curves

BS-C-1 cells grown in 12-well culture plates to near confluency were washed and infected with vIND or vIND-ZIKV (D4W) at an MOI of 5, in triplicate wells, for 1 h. After 1 h, cells were washed and overlaid with D-MEM supplemented with 2.5% FBS and 1 µg/ml DOX. Plates were incubated at 37 °C and at the indicated time points (0 or 24 h), cell lysates were collected and processed. Processed cell lysates were then diluted and added to fresh BS-C-1 cells in 24-well culture plates in duplicate to determine viral titer. Infected plates were stained 2 days later with 0.5% crystal violet in 10% ethanol/20% formaldehyde and plaques were enumerated.

### Safety of vIND-ZIKV in normal mice

Female CB6F_1_/J mice (stock No. 100007, 6 weeks of age, n = 5) were inoculated intranasally with 2 × 10^4^ PFU vIND or vIND-ZIKV (D4W) and weighed daily for 21 days. Mice were given either normal drinking water (NO DOX treatment) or 0.125 mg/ml DOX in the drinking water, replaced every 2 days (DOX treatment).

### Immunogenicity and efficacy of vIND-ZIKV in mice

To assess CMI responses, 6-week-old female C57BL/6J mice (stock No. 000664, n = 5) were inoculated with 10^7^ PFU of vIND, vIND-ZIKV (D4W), or PBS intramuscularly in the right hind limb. Mice were sacrificed after 7 days and spleens were harvested for ELISPOT analysis. To assess humoral immune responses, 6-week-old female C57BL/6J mice (n = 8) were vaccinated intramuscularly with 10^7^ PFU of vIND or vIND-ZIKV (D4W) and sacrificed 4 weeks after vaccination. Blood was collected retro-orbitally on day 0 (naïve sera) or at euthanasia by cardiac puncture for PRNT. To assess humoral immune responses and efficacy, 6-week-old C57BL/6J mice (n = 8) were inoculated with 10^7^ PFU of vIND, vIND-ZIKV (D4W), or PBS intramuscularly. Two weeks later, mice were boosted intramuscularly with 10^7^ PFU of vIND, vIND-ZIKV (D4W), or PBS. Two weeks later, mice were challenged with 10^4^ PFU of ZIKV (strain PRVABC59) intraperitoneally. One day prior to challenge, mice were given 2 mg of anti-IFNAR1 antibody (Leinco Technologies, MAR1-5A3, I-401) intraperitoneally. Mice were bled retro-orbitally on days 0, 14, 27, and 30, and bled via cardiac puncture at euthanasia on day 42. To test the effect of prior vector immunity on the immunogenicity of vIND-ZIKV, 6-week-old female C57BL/6J mice (n = 8) were first primed intramuscularly with 10^7^ PFU vIND or PBS. Two weeks later, mice were vaccinated to begin the humoral immune response and efficacy experiments, exactly as described above.

### ELISPOT assay

First, 96-well ELISPOT plates (mouse IFN-γ ELISPOT set, BD Biosciences, San Jose, CA, USA) were coated with purified anti-mouse IFN-γ overnight at 4 °C. Plates were then blocked for at least 2 h with RPMI 1640 containing 10% FBS and 1 × antibiotic–antimycotic (Thermo Fisher Scientific). Next, 4 µg/ml peptide (BEI Resources, NR-50553, IGVSNRDFVEGMSGG), which during pilot studies was determined to be the most immunogenic among five peptides tested that contained previously-described H-2^b^ E epitopes^41,42^, or 5 ng/ml phorbol myristate acetate (PMA) containing 500 ng/ml ionomycin, was added to the well followed by 2 × 10^5^ freshly harvested splenocytes. The cells were incubated for 18 h at 37 °C. Cell suspensions were aspirated and plates were washed twice with water and three times with PBS-Tween before adding biotinylated anti-mouse IFN-γ. After incubation for 2 h, plates were washed 3 times with PBS-Tween before Streptavidin-HRP was added. After 1 h incubation, wells were washed four times with PBS-Tween, twice with PBS, and BD AEC substrate kit (BD Biosciences) was added. Spot development was monitored and stopped after 15 min by washing wells with water. Plates were air-dried overnight before spots were counted manually after imaging with a stereoscope.

### Plaque reduction neutralization assay (PRNT)

To measure the ability of serum to neutralize ZIKV, PRNTs were performed. Briefly, 12-well cell culture plates were seeded with Vero cells so that they were near confluency at the time of infection. Serum was heat-inactivated at 37 °C for 30 min. Serum samples collected during the immunogenicity study (at euthanasia) were analyzed individually, while serum collected during the efficacy studies (via periodic retro-orbital bleeding) were pooled due to low volumes. Serum was diluted twofold in complete DME containing 1 × antibiotic–antimycotic and mixed with equal volumes of ZIKV strain PRVABC59 containing approximately 50 PFU/well. Serum/virus dilutions were incubated for 1 h at 37 °C, 5% CO_2_. After incubation, cells were infected with the serum/virus dilutions in duplicates for 1 h, inoculum was then aspirated and cells were overlaid with complete DME containing 1 × antibiotic–antimycotic, 2.5% FBS, and 1% methylcellulose. Plates were incubated for 4 days at 37 °C prior to fixation with 0.5% crystal violet in 10% ethanol/20% formaldehyde and manual plaque counting. The PRNT_50_ was calculated as the reciprocal of the highest dilution that resulted in at least 50% reduction in ZIKV plaques.

### ELISA assays

To detect antibodies against ZIKV, serum collected from mice was diluted 1:100, 1:500, 1:1000, 1:4000, or 1:5000 for ELISA. Recombivirus Mouse Anti-Zika Virus Envelope Protein IgG kit (RV-403120-1, Alpha Diagnostic International, San Antonio, TX, USA) and Recombivirus Mouse Anti-Zika Virus NS1 Protein IgG kit (RV-403320-1) were performed according to manufacturer’s instructions. Optical density (OD) was measured at 450 nm with a reference wavelength of 630 nm. Antibody concentrations (U/ml) were calculated based on a standard curve. Lower limit of detection (LLD) was 100 U/ml.

To detect antibodies against VACV, an in-house ELISA was developed. Briefly, flat-bottom 96-well plates were coated with VACV strain WR (~ 2 × 10^4^ PFU) diluted in 100 µl PBS containing 0.1% FBS and incubated overnight at 4 °C. Serum collected from mice was pooled due to low volumes and subsequently serially diluted twofold in PBS containing 5% non-fat milk and 0.05% Tween. Plates were washed and blocked for 1 h in PBS containing 5% non-fat milk and 0.05% Tween. Plates were washed, serial dilutions of serum were added, and plates were incubated for 2 h. Plates were then washed, anti-mouse IgG-HRP conjugate (31430, Invitrogen, Carlsbad, CA, USA) diluted 1:1000 in PBS containing 5% non-fat milk and 0.05% Tween-20 was added, and plates were incubated for 1 h. Plates were washed, TMB Substrate (N301, Thermo Fisher Scientific) was added, the reaction was stopped with 2 M H_2_SO_4_, and OD was measured at 450 nm. Endpoint titers were calculated as the reciprocal of the highest serum dilution that gave a reading above the cutoff (upper prediction limit of a Student *t*-distribution of the no-serum control readings at 95% confidence interval)^43^.

### Analysis of ZIKV viremia by qRT-PCR

Blood was collected by retro-orbital bleeding to analyze ZIKV viremia 2 days after challenge. RNA was extracted from 20 µl mouse serum using the QIAamp Viral Mini Kit (Qiagen, Venlo, Netherlands) per manufacturer’s instructions. The qRT-PCR assays were performed on the RNA samples in triplicate using iTaq Universal Probes One-Step Kit (BioRad) with primers previously described^24^. PFU equivalents were calculated using a standard curve prepared from a previously titrated sample of the ZIKV strain PRVABC59.

### Statistical analyses

Statistical analyses were performed using GraphPad Prism v.7.0e software (GraphPad Software, La Jolla, CA) as described in the figure legends. A p value less than 0.05 was considered statistically significant.

## Supplementary Information

Supplementary Information.

## Data Availability

All data generated or analyzed during the current study are available from the corresponding author on reasonable request.
