# Supplementary material for: Single dose of a replication-defective vaccinia virus expressing Zika virus-like particles is protective in mice
Source: Sci Rep. 2021 Mar 22;11:6492. doi: 10.1038/s41598-021-85951-7 (PMC7985303; doi:10.1038/s41598-021-85951-7)
Supplement: Supplementary file 1 — Supplementary Information. [file 41598_2021_85951_MOESM1_ESM.docx]

**SUPPLEMENTARY INFORMATION**

# Single dose of a replication-defective vaccinia virus expressing Zika virus-like particles is protective in mice

Brittany Jasperse^1^, Caitlin M. O’Connell, Yuxiang Wang, Paulo H. Verardi*

Department of Pathobiology and Veterinary Science and Center of Excellence for Vaccine Research, College of Agriculture, Health, and Natural Resources, University of Connecticut, Storrs, CT 06269, United States

^1^Present address: Department of Microbiology and Immunology, School of Medicine, University of North Carolina at Chapel Hill, Chapel Hill, North Carolina, United States

Correspondence and requests for materials should be addressed to P.H.V. (email: paulo.verardi@uconn.edu)


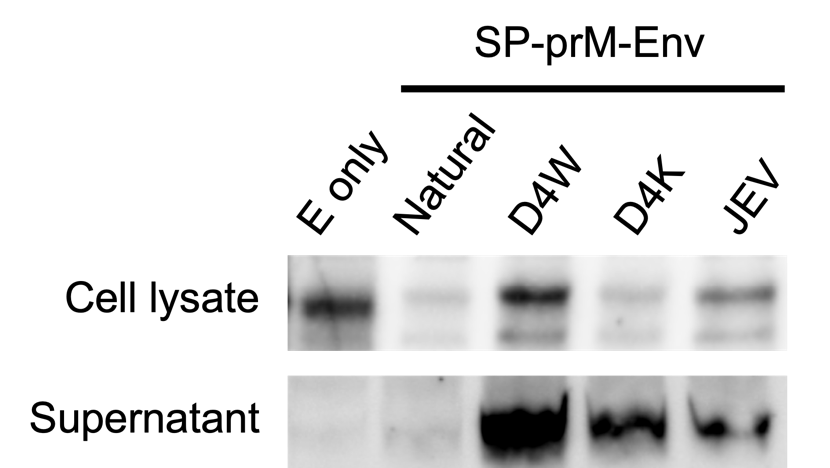


**Supplementary Figure 1.** Western blot of Vero cells (lysates or supernatants) infected with the vaccine candidates in the presence of DOX. Full western blot images are shown in Supplementary Figure 4. Bands of approximately 55 kDa were observed.


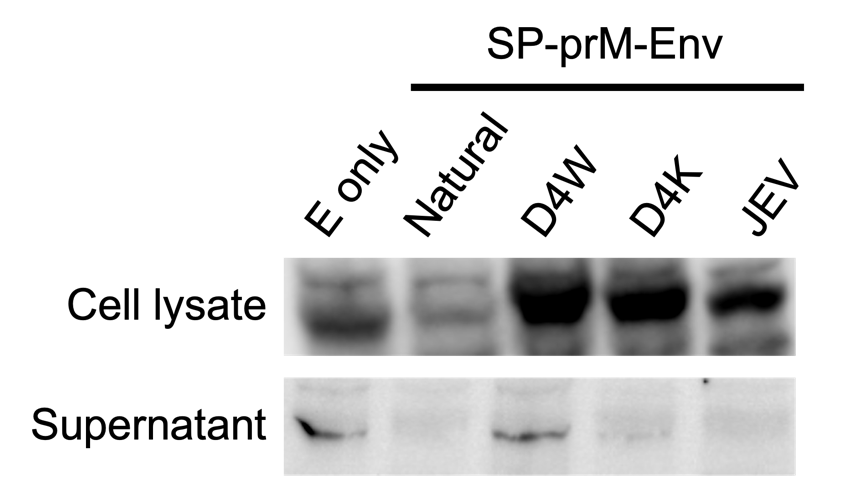


**Supplementary Figure 2.** Western blot of HeLa S3 cells (lysates or supernatants) infected with the vaccine candidates in the absence of DOX. Full western blot images are shown in Supplementary Figure 5. Bands of approximately 55 kDa were observed.

**A**


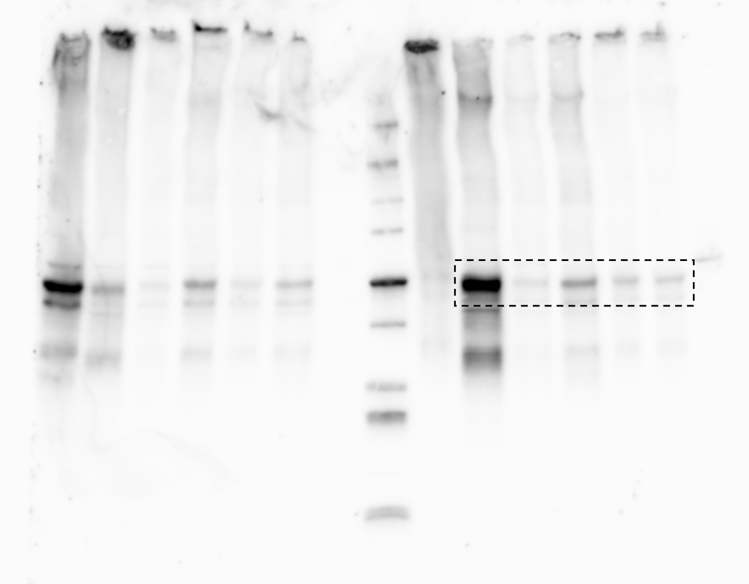


**B**

**Supplementary Figure 3.** Western blot of Vero cells (lysates or supernatants) infected with the vaccine candidates in the absence of DOX. (**A**) Cell lysates of Vero cells infected in the absence of DOX. (**B**) Supernatant of Vero cells infected in the absence of DOX. Complete western blot images are shown. Areas shown in Figure 1, panel b are indicated with dashed rectangles. Protein ladder standards are indicated with arrows.

**A**

**
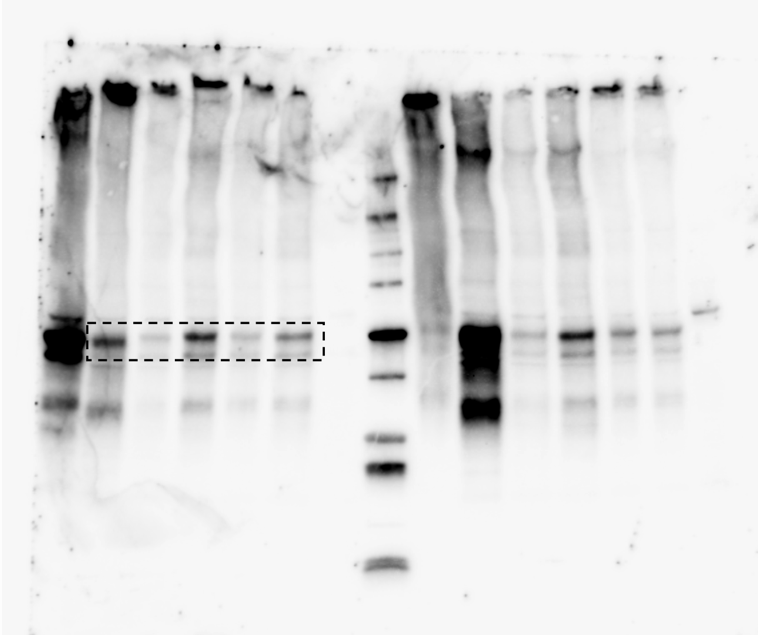
**

**B**

**
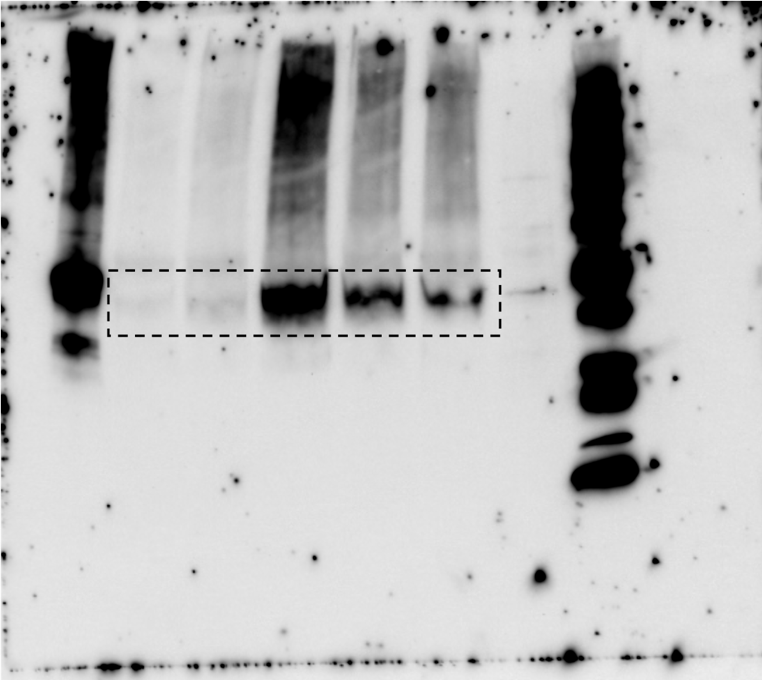
**

**Supplementary Figure 4.** Western blot of Vero cells (lysates or supernatants) infected with the vaccine candidates in the presence of DOX. (**A**) Cell lysates of Vero cells infected in the presence of DOX. Western blot shown is the same shown in Supplementary Figure 3A with increased exposure time. (**B**) Supernatant of Vero cells infected in the presence of DOX. Complete western blot images are shown. Areas shown in Supplementary Figure 1 are indicated with dashed rectangles. Protein ladder standards are indicated with arrows.

**A**


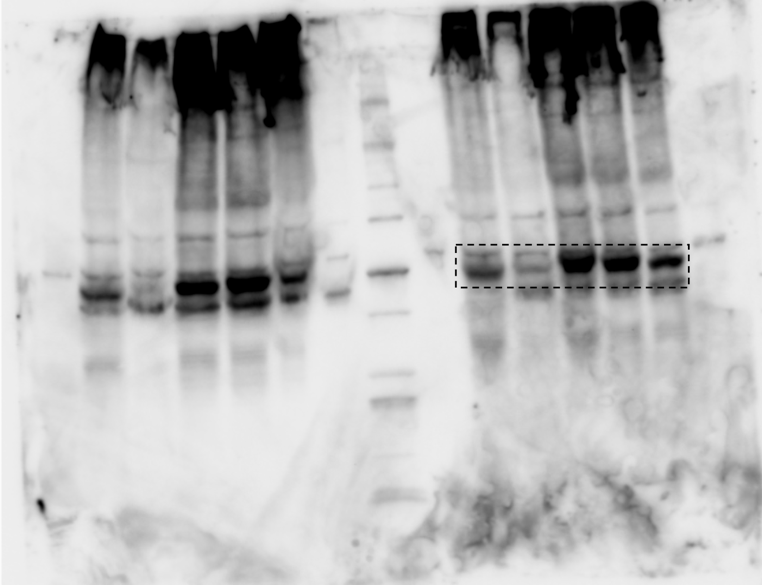


**B**


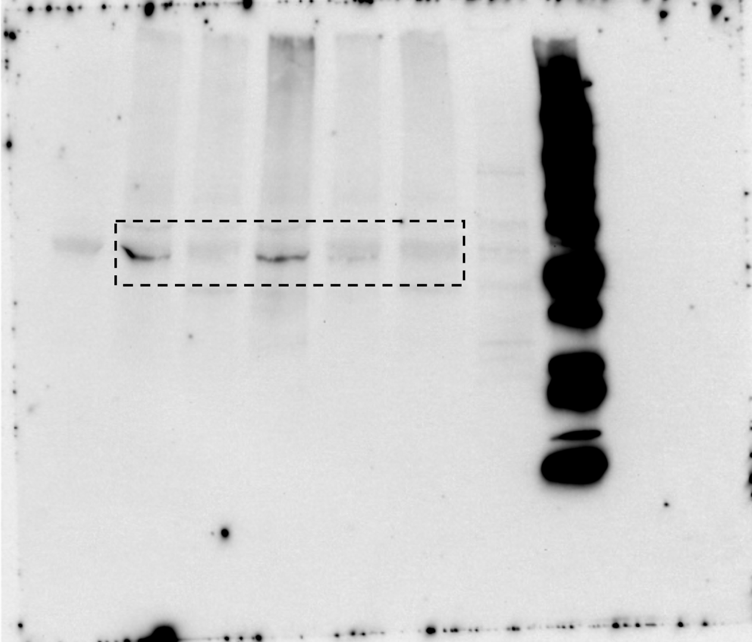


**Supplementary Figure 5.** Western blot of HeLa S3 cells (lysates or supernatants) infected with the vaccine candidates in the absence of DOX. (**A**) Cell lysates of HeLa S3 cells infected in the absence of DOX. (**B**) Supernatant of HeLa S3 cells infected in the absence of DOX. Complete western blot images are shown. Areas shown in Supplementary Figure 2 are indicated with dashed rectangles. Protein ladder standards are indicated with arrows.

| **Primer** | **Sequence (5’–3’)** |
| --- | --- |
| PCR Forward 1 | TACTCGAGATGGGCGCAAAG |
| PCR Forward 2 | ACCTAGCTTCTGGGCGAGTT |
| PCR Forward 3 | GCCCAACACAAGGTGAAGC |
| PCR Reverse 1 | CCAGTGCTTCTTTGTTGTTCC |
| PCR Reverse 2 | TTGTGATGGCAGGTTCCGTA |
| PCR Reverse 3 | CGCGGTTAGTGATGGTGATG |
| Sequencing Forward 1 | GTAAAACGACGGCCAG |
| Sequencing Forward 2 | CCCAAGTTGATGTCGTGTTG |
| Sequencing Forward 3 | TGACCAAGTATATGACTTTTTGGC |
| Sequencing Forward 4 | GCAGCTCTAATGCGCTGTTA |
| Sequencing Reverse 1 | CAGGAAACAGCTATGAC |
| Sequencing Reverse 2 | AACTTAGATTGAAGGGCGTGTC |
| Sequencing Reverse 3 | CCACCATTTGGGGACTCTTA |
| Sequencing Reverse 4 | CCATGATCTGTATATAACAC |

**Supplementary Table 1.** Primer sequences used to authenticate recombinant vaccinia viruses.
